# Robust RT-qPCR Data Normalization: Validation and Selection of Internal Reference Genes during Post-Experimental Data Analysis

**DOI:** 10.1371/journal.pone.0017762

**Published:** 2011-03-15

**Authors:** Daijun Ling, Paul M. Salvaterra

**Affiliations:** Department of Neuroscience, Beckman Research Institute of City of Hope, Duarte, California, United States of America; Naval Research Laboratory, United States of America

## Abstract

Reverse transcription and real-time PCR (RT-qPCR) has been widely used for rapid quantification of relative gene expression. To offset technical confounding variations, stably-expressed internal reference genes are measured simultaneously along with target genes for data normalization. Statistic methods have been developed for reference validation; however normalization of RT-qPCR data still remains arbitrary due to pre-experimental determination of particular reference genes. To establish a method for determination of the most stable normalizing factor (NF) across samples for robust data normalization, we measured the expression of 20 candidate reference genes and 7 target genes in 15 *Drosophila* head cDNA samples using RT-qPCR. The 20 reference genes exhibit sample-specific variation in their expression stability. Unexpectedly the NF variation across samples does not exhibit a continuous decrease with pairwise inclusion of more reference genes, suggesting that either too few or too many reference genes may detriment the robustness of data normalization. The optimal number of reference genes predicted by the minimal and most stable NF variation differs greatly from 1 to more than 10 based on particular sample sets. We also found that *GstD1*, *InR* and *Hsp70* expression exhibits an age-dependent increase in fly heads; however their relative expression levels are significantly affected by NF using different numbers of reference genes. Due to highly dependent on actual data, RT-qPCR reference genes thus have to be validated and selected at post-experimental data analysis stage rather than by pre-experimental determination.

## Introduction

Real-time polymerase chain reaction (PCR) combined with reverse transcription (RT-qPCR) has been widely used for quantification of gene expression that may associate with specific biomedical conditions. However, RT-qPCR measures the mRNA transcript levels differentially contributed by specific biological conditions as well as confounding factors that are non-specific to the biological conditions and non-reproducible in different experiments. Even with careful control of technical variables [1], [2], [3], confounding factors may still result from sample-to-sample and run-to-run variations particularly in RNA extraction and reverse transcription efficiency, random pipetting errors, etc. Data normalization using internal reference genes is thus a crucial step necessary to minimize the influence of confounding factors and improve the fidelity of the quantification process with respect to the specific biological conditions. The internal reference genes pass through all steps of the analyses simultaneously along with target genes and should thus minimize the confounding variations among parallel samples. What and how many reference genes used for calculation of normalization factors (NF) in parallel samples is thus a crucial determinant of the accuracy of expression quantification.

Internal reference genes are usually chosen from “housekeeping” genes with abundant and stable expression under various experimental conditions [4]. In current applications, however, RT-qPCR quantification remains problematic [4], [5], [6] due to arbitrary determination of the number and selection of particular reference genes for data normalization. Most frequently only a single reference gene is used for data normalization. Even though robust statistic methods have been developed for evaluation of multiple reference genes [7], [8]; the selection of particular genes or the number of reference genes remains unchanged in different experiments. In addition, the relationship between the number of reference genes and the accuracy in RT-qPCR data normalization has not been clearly addressed. Here we investigate these issues using a panel of 20 candidate reference genes and 15 cDNA samples from *Drosophila* heads that are associated with brain aging or neurodegeneration.

The fruit fly, *Drosophila melanogaster*, constitutes a valuable model organism for aging research and is becoming increasingly popular for the study of neurodegeneration. Quantitative examination of gene expression in *Drosophila* brains during aging may help to identify the genetic components of neuronal aging as well as genetic modifiers of neurodegenerative diseases. Even though RT-qPCR is a powerful tool to achieve this goal, a systematic verification of expression stability for reference genes used for RT-qPCR data normalization is still absent in *Drosophila*. The so-called “housekeeping” genes most often selected for normalization of transcription variation in *Drosophila* tissues are adopted from other species without experimental verification. In many cases, however, the expression stability of these genes in other species is also problematic. In this study, we measured the expression stability of 20 candidate reference genes most of which have been previously used as typical PCR reference genes. We found that their expression stability varies among different sample subsets. No particular gene exhibits constant expression stability among various samples negating its suitability for all-purpose data normalization. Accurate data normalization thus requires an experiment-specific subset of internal reference genes selected from a particular gene panel and optimized for a particular sample set.

## Results

### PCR efficiencies and Ct profiling of candidate reference genes

**Figure 1:**
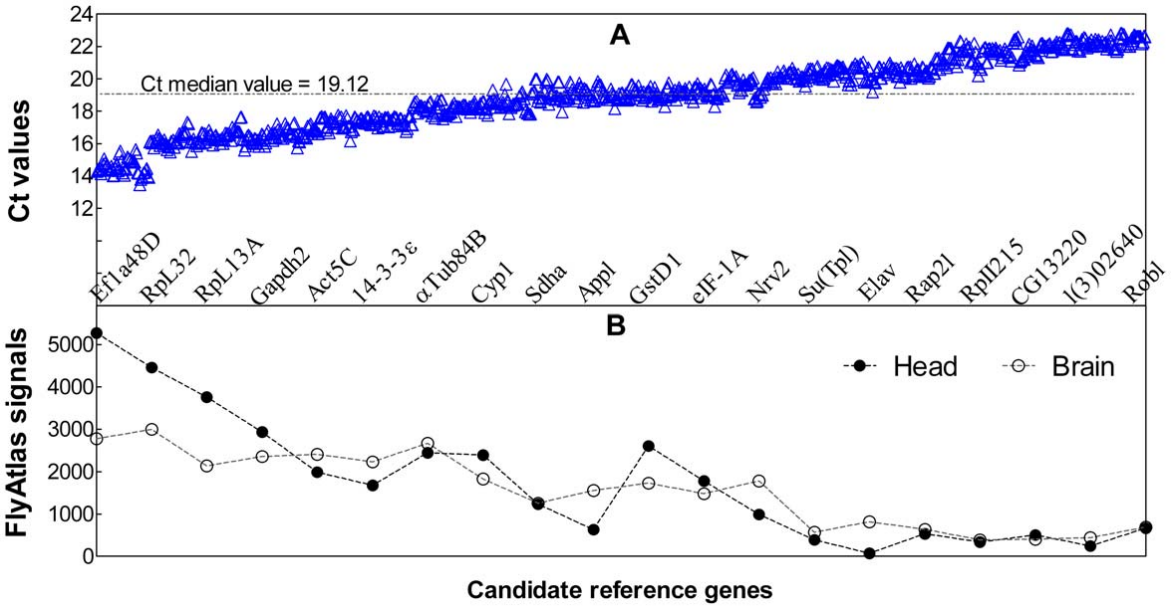
Ct values of 20 candidate reference genes and their mRNA levels reported in the FlyAtlas. (A) Scatter plot of the raw Ct values obtained from the 9 aging-related cDNA samples. Each gene has an average of 54 Ct values and the order of genes has been sorted by the mean Ct values. The Ct values were adjusted to the same baseline using IQ5 software (Bio-Rad) for construction of this plot and are thus not exactly the same as those used in other statistical analyses. (B) The reported mRNA levels from FlyAtlas for the 20 candidate reference genes. The FlyAtlas microarray signals are negatively correlated to the Ct values (Ct vs. FlyAtlas brain: Pearson's R = −0.905, P<0.0001; Ct vs. FlyAtlas head: Pearson's R = −0.882, P<0.0001).

Genome-wide expression of most *Drosophila* genes has been measured previously in multiple tissues of 7-day-old adults of Canton-S strain using Affymetrix microarray and is publicly accessible in the FlyAtlas expression database [9]. In order to avoid high Ct values which could result in irreproducible RT-qPCR quantification [10], [11], we excluded candidate reference genes with low expression in fly brain/heads (FlyAtlas values<100). The linear regression for 10-fold dilution series of standard samples shows that the squared correlation coefficients (R^2^) of all tested primer sets are greater than 99%. The primer sets for *Exba* and *Faf* have lower PCR efficiencies (93 and 83% respectively) and were excluded in this study (Table S2). The raw Ct values for the remaining 20 genes measured in 9 aging-related samples range from 13.5–22.8 (Fig. 1A) which is acceptable for reliable RT-qPCR quantification. The Ct values correlate with expression levels derived from mRNA microarray signals reported in FlyAtlas (Fig. 1B).

### Candidate reference genes exhibit sample-specific expression stability

**Figure 2:**
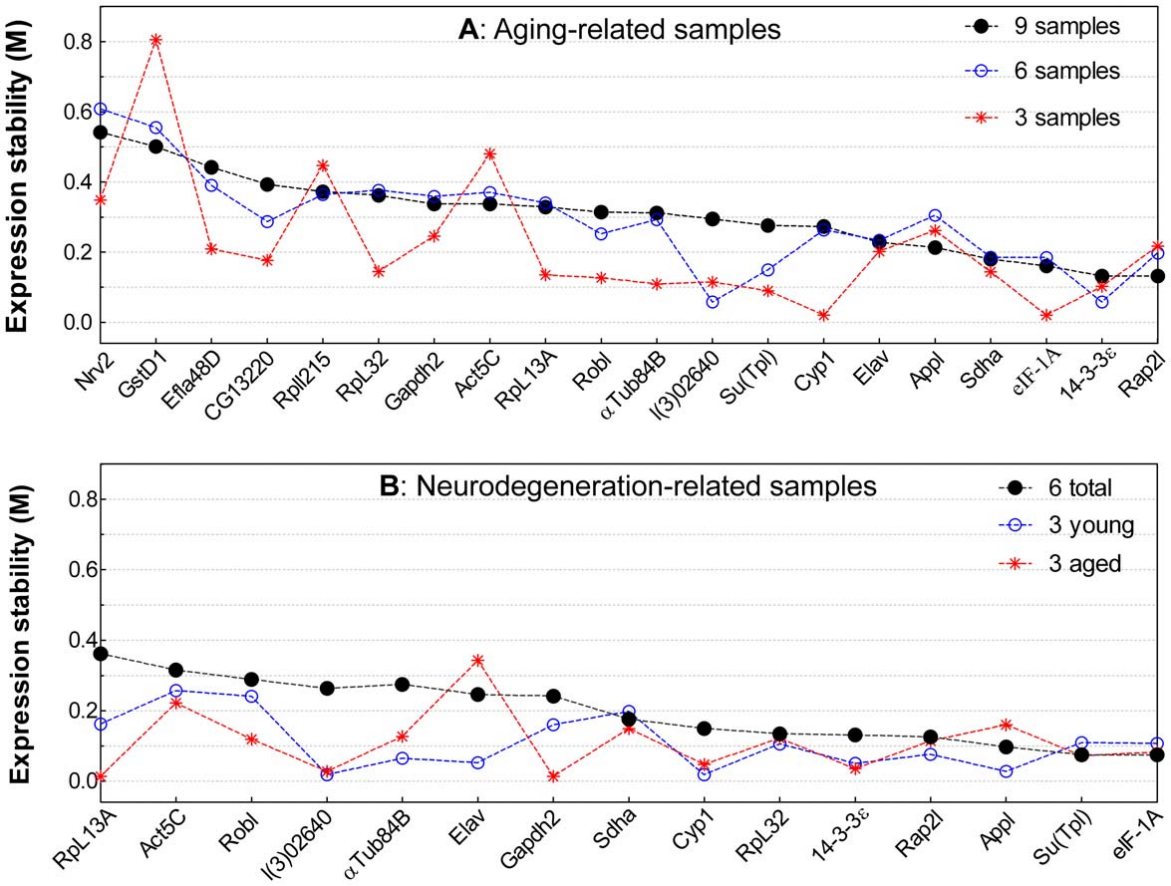
Expression stability (M value) of candidate reference genes in different sample subsets. (A) M values for 20 candidate reference genes calculated across different aging-related sample subsets (3 samples: control, mid- and old-age; 6 samples: the previous 3 samples plus low-T, high-T and male samples; 9 samples: the previous 6 samples plus 3 additional treatments. See Table S3 for details). (B) M values of the 15 candidate reference genes calculated across different neurodegeneration-related sample subsets (3 young: control, Aβ_1–42_ and tau at 3 days; 3 aged: control, Aβ_1–42_ and tau at 20 days; 6 total: combined 3 young and 3 aged samples. See Table S3 for details).

To determine the best RT-qPCR reference genes from the gene panel with 20 candidates, we evaluated their expression stability across 15 aging- or neurodegeneration-related samples (Table S3). Expression stability for a particular gene is reflected by the M value calculated as the mean standard deviation of the log-transformed expression ratios across samples for the particular gene relative to other reference genes remaining in the gene panel [7]. The calculation was performed by stepwise exclusion of individual gene with the highest M value (i.e. the least stable gene) from the panel until reaching the last two genes with the smallest M value (i.e. the most stable genes). The M values for the 20 candidate genes were first evaluated across 9 aging-related samples (Fig. 2A). We excluded the 5 least-stable genes (*Nrv2*, *GstD1*, *Efla48D*, *RpII215* and *CG13220*) and the remaining 15 genes were further evaluated in 6 neurodegeneration-related samples (Fig. 2B). Previous studies defined M<1.5 as an acceptable criterion for selection of RT-qPCR reference genes [12], [13]. In our samples, the M values for all genes are less than 1.0.

The expression stability of the candidate reference genes exhibits obvious discrepancies when compared in different sample subsets. For example, the expression stability of *Act5C* in the aging-related samples has a rank order of 13 (from most to least stable) when calculated across 9 samples, but is 19 when calculated across 3 samples (Fig. 2A). This type of discrepancy is even more apparent in different neurodegeneration-related sample subsets. *Elav* and *Appl*, for example, show relatively good stability in young samples but poor stability in aged samples (Fig. 2B). *RpL13A* exhibits the least stability when calculated across the 6 neurodegeneration-related samples but is one of the most stable genes when calculated across the 3 aged samples. In addition, the most stable genes with the lowest M values calculated in each sample subset are not exactly the same. This result suggests that the expression stability of a particular gene is not constant in different sample sets even if all samples have the same tissue composition. The expression stability of candidate reference genes is thus sample specific or more precisely analysis specific.

### V_n/n+1_ curve and the optimal number of reference genes for data normalization

Proper normalization of expression data across samples determines the accuracy of RT-qPCR quantification. To obtain the optimal number of reference genes for data normalization, we calculated the pairwise variation (V_n/n+1_) of serial log-transformed NF ratios using N relative to N+1 reference genes (i.e. log_2_(NF_n_/NF_n+1_) as previously described [7]). The V_n/n+1_ value reflects NF stability across samples. While individual reference genes have considerably differential expression levels across samples, NF will be sensitive to stepwise inclusion of these reference genes resulting in an increase or decrease in V_n/n+1_ value. If inclusion of more or less reference genes has little or no effect on V_n/n+1_ value, NF will become insensitive to stepwise inclusion of these reference genes and approach a relatively stable status with a minimal V_n/n+1_ value. The corresponding number of validated reference genes will approach the most reliable NF calculation across sample leading to accurate data normalization.

**Figure 3:**
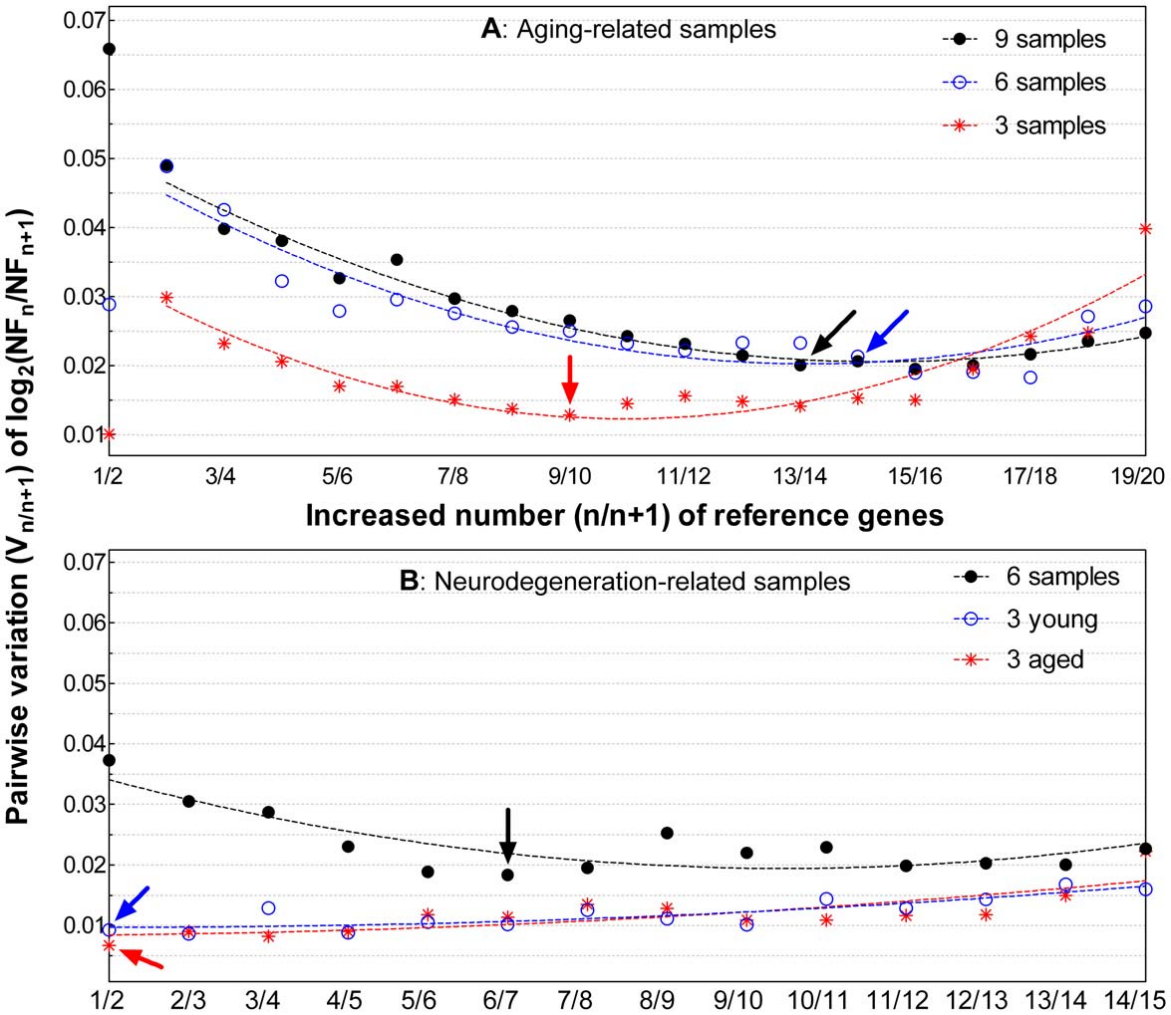
Pairwise variation (V_n/n+1_) of NF ratios across samples and the optimal number of reference genes. (A) The V_n/n+1_ of 20 candidate reference genes calculated across different numbers of aging-related samples. (B) The V_n/n+1_ of 15 candidate genes calculated across different numbers of neurodegeneration-related samples. Stepwise inclusion of individual genes is based on their rank order of expression stability using indicated sample subsets (Fig. 2). The data points were fit well with the second order polynomial curves. The arrows pointing the minimal V_n/n+1_ value indicate the optimal numbers of reference genes for indicated sample subsets.

The V_n/n+1_ calculated across the 9 aging-related samples exhibits an initial decrease as stepwise inclusion of individual reference genes (Fig. 3A, black line), suggesting that more (>5) reference genes likely achieve more stable NF across samples. Subsequent inclusion of additional reference genes make V_n/n+1_ slowly approach a minimal value. An interesting finding, however, is that continuous addition of more reference genes (>17, Fig. 3A, black line) results in an increase in V_n/n+1_. This increased NF variation is likely due to the stepwise inclusion of genes with relatively unstable expression. This broad U-shape curve suggests that inclusion of either too few or too many reference genes may detriment the robustness of data normalization.

The minimal V_n/n+1_ on the U-shape curve represents the most stable NF achievable within a particular sample set and a particular panel of reference genes, thus corresponding to the optimal number of reference genes for the most accurate data normalization. The optimal number of reference genes varies when calculated among different sample subsets. For the aging-related samples and 20 input reference genes, the optimal number is 13 when calculated across 9 samples, 14 across a subset of 6 samples and 9 across a subset of 3 samples (Fig. 3A, arrows). Similar analyses among subsets of the neurodegeneration-related samples and 15 input reference genes show that 6 reference genes are optimal for the total 6 samples while only a single reference gene is optimal for either the 3 young or the 3 aged samples (Fig. 3B, arrows). The shape of the V_n/n+1_ curves suggests that the optimal number of reference genes for reliable RT-qPCR data normalization should be determined by stepwise inclusion of individual reference genes based on their expression stability until a relatively stable and minimal NF variation is achieved across samples in a particular assay.

### Target gene expression normalized by different numbers of reference genes

**Figure 4:**
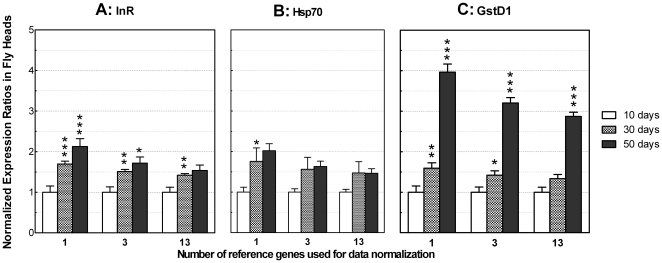
Relative expression of target genes normalized with different numbers of reference genes. (A–C) Target genes expressed in fly heads at 30 or 50 days relative to 10 days. The relative expression levels were normalized by 1, 3 or 13 validated reference genes: *Rap2l* (1 reference gene); *14-3-3e*, *Rap2l* and *eIF-1A* (3 reference genes); *14-3-3e*, *Act5C*, *Appl*, *Cyp1*, *Elav*, *Rap2l*, *Robl*, *RpL13A*, *Sdha*, *Su(Tpl)*, *αTub84B*, *eIF-1A* and *l(3)02640* (13 reference genes). The reference genes are selected based on the rank order of expression stability for the 20 candidate reference genes in the 9 aging-related samples (Fig. 2A, black line). Data are mean+SEM. *, P<0.05; **, P<0.01; ***, P<0.001. Two-tailed P values were calculated between indicated column and its left adjacent column using Student's t-test. (A) *InR* expression exhibits an age-dependent increase; however, normalization with 1, 3 or 13 reference genes results in a sequential decrease in both the magnitude and statistical significance of expression differences across age groups. (B) The age-dependent increased *Hsp70* expression also shows minor decrease when normalized sequentially by 1, 3 or 13 reference genes. (C) An age-dependent increase in relative expression of *GstD1*.

Normalization of RT-qPCR data has been performed most frequently using either a single reference gene or 3 reference genes as a proposed way to increase accuracy. However, we show here that the optimal number of reference genes for RT-qPCR data normalization may change from analysis to analysis. Arbitrary selection of more or fewer reference genes may thus decrease the accuracy of calculating target gene expression. For an example study, here we measured the transcript variations of 7 target genes (Table 1) in 9 aging-related samples using 1, 3 or 13 reference genes for data normalization. The relative levels of *Atg1*, *CathD* and *Rab5* expression show no significant differences when calculated using the different numbers of reference genes (Table S4). However, *InR* expression, when normalized by only 1 reference gene, exhibits a significantly age-dependent increase (Fig. 4A). Normalization using 3 reference genes lowers the magnitude of relative expression level but the trend of the age-dependent increase is still apparent. When data are normalized by 13 reference genes, the optimal number predicted for this sample group (Fig. 3A), the expression level in the 50-day samples is not significantly different from that in the 30-day samples. After using optimal number of reference genes for data normalization, the modified conclusion is that brain *InR* expression in older flies is higher than in young flies but does not show significantly age-dependent increase. Similar changes also occur in the calculated relative expression levels of *Hsp70* and *GstD1* after normalization by 1, 3 or 13 reference genes respectively (Fig. 4B and 4C). Note that all reference genes are selected on the basis of their rank ordered expression stability (Fig. 2A). The relative expression levels of these genes are even more divergent if calculated using arbitrarily selected reference genes (for example, *Act5C*, data not shown). These results indicate that appropriate calculation of NF across samples determines both the magnitude of relative expression levels and its statistical significance. Thus determination of the optimal number of reference genes is important for accurate normalization of RT-qPCR data especially when differences in expression levels are subtle.

**Table 1:** Candidate reference and target genes used in this study.

*Drosophila* gene			Human homologue	References
Symbol	Name	Locus ID		
**22 candidate reference genes**				
*Gapdh2*	Glyceraldehyde-3-phosphate dehydrogenase	CG8893	*GAPDH*	[4], [7], [13], [14]
*αTub84B*	Alpha tubulin	CG1913	*TUB*	[9], [13], [14], [18]
*RpL32*	Ribosomal protein 49/L32	CG7939	*RP49*	[9], [13], [14]
*RpL13A*	Ribosomal protein L13a	CG1475	*RPL13a*	[7], [14], [18]
*Ef1α48D*	Elongation factor 1 alpha	CG8280	*EF1a*	[13]
*eIF-1A*	Eukaryotic initiation factor 1A	CG8053	*EIF1AY*	[9]
*Sdha*	Succinate dehydrogenase A	CG17246	*SDHA*	[4], [7], [14]
*GstD1*	Glutathione-S-transferase 1	CG10045	*GST1*	[14]
*Cyp1*	Peptidylprolyl isomerase F	CG9916	*PPIF*	
*14-3-3ε*	Tyrosine-3-monooxygenase	CG31196	*YWHAE*	[4], [7], [9]
*exba*		CG2922	–	[9]
*Act5C*	Actin	CG4027	*ACTG1*	
*Su(Tpl)*	elongation factor RNA polymerase II	CG32217	*ELL*	[9]
*Faf*	Fas-associated factor	CG10372	*FAF2*	[9]
*CG13220*		CG13220	–	[9], [13]
*robl*	dynein, light chain, roadblock-type 2	CG10751	*DYNLRB2*	[9]
*Rap2l*	Ras-associated protein 2-like	CG3204	*RAP2B*	
*l(3)02640*	hydroxymethylbilane synthase	CG9165	*HMBS*	[4], [7], [14], [18]
*RpII215*	RNA polymerase II	CG1554	*RPII*	[4], [18]
*nrv2*	Na^+^/K^+^ ATPase	CG9261	*ATP1B2*	
*Elav*	Embryonic lethal abnormal vision	CG4262	*ELAVL2*	[20], [23]
*Appl*	Beta amyloid protein precursor-like	CG7727	*App*	[19], [24]
**7 target genes**				
*Atg1*	Autophagy-specific gene 1	CG10967	*ULK2*	
*Rab5*	Rab-protein 5	CG3664	*RAB5A*	
*Lamp1*	lysosomal-associated membrane protein 1	CG3305	*LAMP1*	
*CathD*	Cathepsin D	CG1548	*CTSD*	
*InR*	Insulin-like receptor	CG18402	*IGF1R*	
*Ire-1*	Inositol-requiring enzyme-1	CG4583	*ERN1*	
*Hsp70*	Heat shock protein-70	CG31366*	*HSPA1*	

*There are multiple *Hsp70* genes in the *Drosophila* genome. The primer set for this gene (Table S2) does not distinguish the RNA transcripts from different *Hsp70* genes including CG31366, CG18743, CG6489, CG31449, CG31359 and CG5834.

We show that stepwise inclusion of more reference genes across 3 aged neurodegeneration-related samples exhibit no apparent NF variation (Fig. 3B, red line), suggesting that a single reference gene is sufficient for data normalization across these samples. To test if there is any significant difference in relative expression normalized between single and multiple reference genes, we normalized the relative expression of the 7 target genes by a single reference gene (*Gapdh2*) or multiple reference genes (*Gapdh2*, *RpL13A* and *l(3)02640*) based on their rank ordered expression stability for 15 candidate reference genes. The results show no obvious differences among 7 target genes in relative expression levels using either 1 or 3 stable reference genes for normalization (Table S5). Thus a single reference gene in this case is sufficient for RT-qPCR data normalization, consistent with the relative stability and low level of the V_n/n+1_ curve starting from the initial part (Fig. 3B, red line). Taken together, what and how many reference genes are sufficient for RT-qPCR data normalization varies on a case-by-case basis. Thus accurate data normalization needs assessment of a panel of candidate reference genes for a particular sample set.

## Discussion

RT-qPCR quantification requires data normalization by internal reference genes that are measured simultaneously along with target genes to offset experimental confounding variations. However, improper selection of reference genes will result in inaccurate calculation of NF and consequently obscure actual biological differences among samples. Here we evaluate the expression stability of 20 candidate reference genes in 15 *Drosophila* head cDNA samples associated with brain aging or Aβ_1–42_/tau-induced neurodegeneration. Although most of these candidates are considered to be “typical” housekeeping genes and are widely used for data normalization, they exhibit considerable variation in expression stability across various sample sets. Pairwise analyses of NF variation through stepwise inclusion of an increasing number of reference genes reveals that the optimal number varies from 1 to more than 10 for a particular sample set. Our results suggest that no particular gene exhibits constant expression stability across all sample sets thus precluding selection of an all-purpose reference gene for data normalization.

The expression stability of candidate reference genes is often estimated using geNorm first developed by Vandesompele, et al in their landmark paper where reference genes with M<1.5 were suggested as appropriate [7]. This cut-off value has been widely adopted as a criterion for selection of reference genes [10], [11], [12], [13], [14], [15]. Our results suggest that adoption of a fixed cut-off M value may be arbitrary since M values change with the composition not only of particular cDNA samples but also of individual reference genes. The individual M values for a particular gene panel are calculated using the same sample set but different subsets of candidate genes due to stepwise exclusion of one gene in each calculation cycle. Our data suggest that the order of stepwise exclusion, rather than the absolute M value, is a better index of the relative expression stability of individual candidate genes within a gene panel. The actual M value is less meaningful and basically incomparable through intra- and inter-analyses. For example, *αTub84B* has a higher M value but its expression is more stable than *l(3)02640* in the 6 neurodegeneration-related samples (Fig. 2B, black line). In terms of the underlying mathematics, the M value of *l(3)02640* was calculated using a gene panel containing 12 candidates and its value (M = 0.263) is the highest among the genes in this panel. The next calculation excluding *l(3)02640* and using a gene panel with the 11 remaining candidates results in *αTub84B* to have the next highest M value (M = 0.274). Thus the M values of *l(3)02640* and *αTub84B* genes are not comparable to each other due to the calculation using different gene panels (12 vs. 11 candidate genes). In another example, the M value of *GstD1* is 0.5 across the 9 aging-related samples (Fig. 2A), obviously less than the arbitrary criterion of 1.5. However, its expression at 50 days is 3 or more times higher than the control samples at 10 days (Fig. 4C). Apparently *GstD1* in aging-related samples is not suitable for use as a valid reference gene since normalization by this gene will obviously result in considerable underestimation of target gene expression in the old age samples.

Another important but underappreciated issue in RT-qPCR quantification is the relationship between NF stability and the number of reference genes used for NF calculation. Most previous studies evaluated fewer than 10 candidate genes that appear to be not sufficient to characterize the relationship between the NF and the number of reference genes. Using 20 candidate reference genes, we see a wide U-shaped relationship between NF stability and the number of reference genes. Characterization of the NF stability across samples is practically important to determine the optimal number of reference genes for data normalization. In previous studies, a cut-off of V_n/n+1_<0.15 was suggested as an appropriate selection criterion for estimating the optimal number of reference genes; or practically 3 stable reference genes were suggested to be sufficient for data normalization in most cases [3], [6], [13]. These suggestions also appear arbitrary without proper statistical verification. For example some analyses had never achieved V_n/n+1_<0.15 but they could obtain the lowest V_n/n+1_ value [6]. Based on the relationship between NF and the number of reference genes characterized here, we propose that the optimal number of reference genes corresponds to the most stable NF achievable with a particular panel of candidate reference genes and a particular sample set.

In summary, 20 candidate genes were evaluated to determine the optimal internal reference genes for RT-qPCR data normalization using *Drosophila* head cDNA samples associated with brain aging or neurodegeneration. The expression stability of the candidate genes exhibits sample-specific variation. The optimal number of reference genes for accurate data normalization is determined by the stable and minimal NF variation achievable in a particular panel of candidate genes and a particular sample set. An improper selection of reference genes for normalization of RT-qPCR data may cause false results. We found that *GstD1*, *Hsp70* and *InR* genes exhibit significantly increased mRNA transcript levels with advancing age. Additional examples appearing in our recent publication addressed the practical application in laboratories of assay-specific selection of internal reference genes and NF optimization for RT-qPCR data analyses [16]. Taken together, RT-qPCR quantification requires the simultaneous measurement of a panel of candidate reference genes rather than few empirically-determined or pre-validated reference genes and that robust data normalization needs to be optimized for each particular assay.

## Materials and Methods

### Candidate genes

Candidate reference genes were selected from a total of 29 “housekeeping” genes that are often used for PCR normalization in other species [4], [7], [13], [14], [17], [18] or were predicted to be stably expressed in various *Drosophila* tissues on the basis of tissue-specific mRNA microarray data [9]. Ten of these genes were not included in this study due to their unsuitability as described (Table S1). An additional 3 *Drosophila* genes (*Elav*, *Appl* and *Nrv2*) were also selected because they are constitutively expressed in *Drosophila* neurons and thought to be neuronal “housekeeping” genes [19], [20]. We evaluated a total of 22 candidates using RT-qPCR tested in various *Drosophila* head cDNA samples. Two genes (*Exba* and *Faf*) were excluded from further experimental and statistical analyses because of lower PCR efficiencies (<95%). Twenty candidate reference genes were included for further experiments and statistical analyses (Table 1). We also studied 7 target genes (Table 1) to determine if their expression levels are associated with brain aging or neurodegeneration conditions.

### Primer design and verification

The common sequences from genes with multiple mRNA transcript variants were used for PCR primer design. Primers were designed using Beacon Designer (Premier Biosoft International). The primer sets with any secondary structures predicted to form primer dimers were excluded. The PCR specificity for each primer set was theoretically verified by Primer-Blast (http://www.ncbi.nlm.nih.gov/tools/primer-blast/) using the *Drosophila* transciptome. Some primer sets were designed to cross exon-intron boundaries. The size of PCR amplicons were limited to 60–250 bp. Additionally, all primer sets were verified to produce a single symmetrical amplicon peak in melting curve analyses and no primer-dimer peaks in no-template-control (NTC) reactions (Fig. S1A–C). Agarose gel electrophoresis was used to experimentally verify PCR specificity and also to rule out any potential contamination of genomic DNA in cDNA samples (Fig. S1D). PCR efficiency was calculated from 10-fold serial dilutions of standard cDNA samples and only primer sets with PCR efficiencies≥95% were accepted (Table S2).

### Fly head samples

*Drosophila melanogaster* were harvested within 24 hours after eclosion and incubated at various conditions (Table S3) in fresh food vials. Live flies were transferred to new food vials every 3 days. Aging-related head samples were from *w^1118^* female flies collected at 10 (Control), 30 (Mid-age) or 50 (Old-age) days after eclosion to model normal neurological aging based on expected lifespan [21]. Ten day old flies were treated with instant starvation, oxidative conditions or heat shock to induce aging-related stressful conditions. An additional 3 samples were incubated at 18°C (Low-T), 25°C (Control) or 32°C (High-T) for 10 days to evaluate temperature-sensitive variations associated with fly lifespan. Neurodegeneration-related samples were from 3 (young samples) or 20 (aged samples) day old female flies with or without expression of neurodegeneration-associated human amyloid beta 42 (UAS-Aβ_1–42_) or tau (UAS-tau^R406W^) whose expression was controlled by a pan-neuronal Elav-Gal4 driver. Aβ_1–42_ and phosphorylated tau are aggregate-prone proteins associated with Alzheimer's disease [21], [22]. Fly samples were snap frozen on dry ice and stored at −80°C prior to RNA extraction. Three biological replicates were used for each experimental condition.

### RNA extraction and RT-qPCR

Total RNA was extracted from 30 fly heads using RNA STAT-60 (Tel-Test), treated with DNase I (Ambion) to remove potential genomic DNA contamination and purified using RNeasy Mini Kit (Qiagen). The integrity of the representative RNA samples was assessed using Agilent 2100 Bioanalyzer (Fig. S1E). Total RNA concentration was measured in duplicate using NanoDrop ND-1000 Spectrophotometer and the purity of the samples was estimated by the OD ratios (A_260_/A_280_, ranging within 1.8–2.2). cDNA was synthesized from 1 µg of DNA-free total RNA in a 20 µl reaction volume using RETROscript Kit (Ambion) and random decamers as reverse transcription primers. cDNA samples were diluted 10-fold for real-time PCR reactions. Gene-specific transcription levels were determined in a 20 µl reaction volume in duplicate using SYBR Green and an IQ5 real-time PCR machine (Bio-Rad) following the manufacturer's instructions. Standard cDNA samples with 10-fold serial dilutions were used for PCR efficiency calculations. Real-time PCR reactions of the standard, test cDNA samples and no template controls (NTC) using the same primer set were analyzed together in the same 96-deep well plate (Bio-Rad) in order to minimize run-to-run variations and use exactly the same threshold setting (user defined baseline subtracted curve fit) for determination of the threshold cycle values (Ct). Parallel samples were processed using the same batch of reagents to minimize overall sample-to-sample variations.

### Data analyses

After completing each real-time PCR run, outlier data points were identified and excluded manually using IQ5 software (Bio-Rad) based on obvious deviations in both the normal shape of amplification curves and the Ct values of other repeated observations (biological triplicates×PCR duplicates). Data analyses were performed using a custom SAS macros to automatically calculate the key variables including PCR efficiency (E) and squared correlation coefficients (R^2^) of primer sets, expression stability (M values) of candidate reference genes, pairwise variation of NF ratios using different numbers of multiple reference genes (V_n/n+1_), normalized expression ratios of target genes and statistic comparison using Student's t-test. The SAS code for calculation of M value and V_n/n+1_ were developed primarily based on the previous algorithm [7].

## Supporting Information

Figure S1 **Quality control of RT and real-time PCR performance.** (A) Representative melting curve for real-time PCR products exhibits only one symmetrical peak. (B) An asymmetric melting curve suggesting unspecific amplification. These types of primer sets were not used for analyses. (C) Absence of a melting peak in no-template-control (NTC) reactions suggesting an absence of primer-dimers during real-time PCR reaction. (D) Agarose gel electrophoresis to verify PCR specificity of the tested primer set and confirm the absence of potential genomic DNA contamination in cDNA samples. The representative data were from the *RpII215*-specific primer set predicted to generate a 308 bp fragment from genomic DNA and a 244 bp fragment from cDNA. Lanes from left to right are: 1, 50 bp DNA ladder (#N3236S, NEB); 2, a genomic DNA sample; 3–6, representative cDNA samples used in our experiments; 7, a negative control without adding M-MLV reverse transcriptase during RT; 8, a cDNA sample without DNase 1 pre-treatment. (E) The integrity of the representative RNA samples were assessed using Agilent 2100 Bioanalyzer. The electropherogram shows 2 sharp rRNA peaks that migrate close to each other, representing a typical *Drosophila* ribosomal RNA profile and suggesting excellent integrity of the RNA sample.
(TIF)

Table S1 **Classic reference genes unsuitable for brain aging and neurodegeneration study in** ***Drosophila*** *.
(DOC)

Table S2 **Sequences and PCR efficiencies of primer sets.**
(DOC)

Table S3 **Experimental details of fly head samples.**
(DOC)

Table S4 **Relative expression of target genes in aging-related samples normalized by different subsets of reference genes.**
(DOC)

Table S5 **Relative expression of target genes in neurodegeneration-related samples normalized by different subsets of reference genes.**
(DOC)
