# Supplementary figures and images for: Robust RT-qPCR Data Normalization: Validation and Selection of Internal Reference Genes during Post-Experimental Data Analysis
Source: PLoS One. 2011 Mar 15;6(3):e17762. doi: 10.1371/journal.pone.0017762 (PMC3058000; doi:10.1371/journal.pone.0017762)

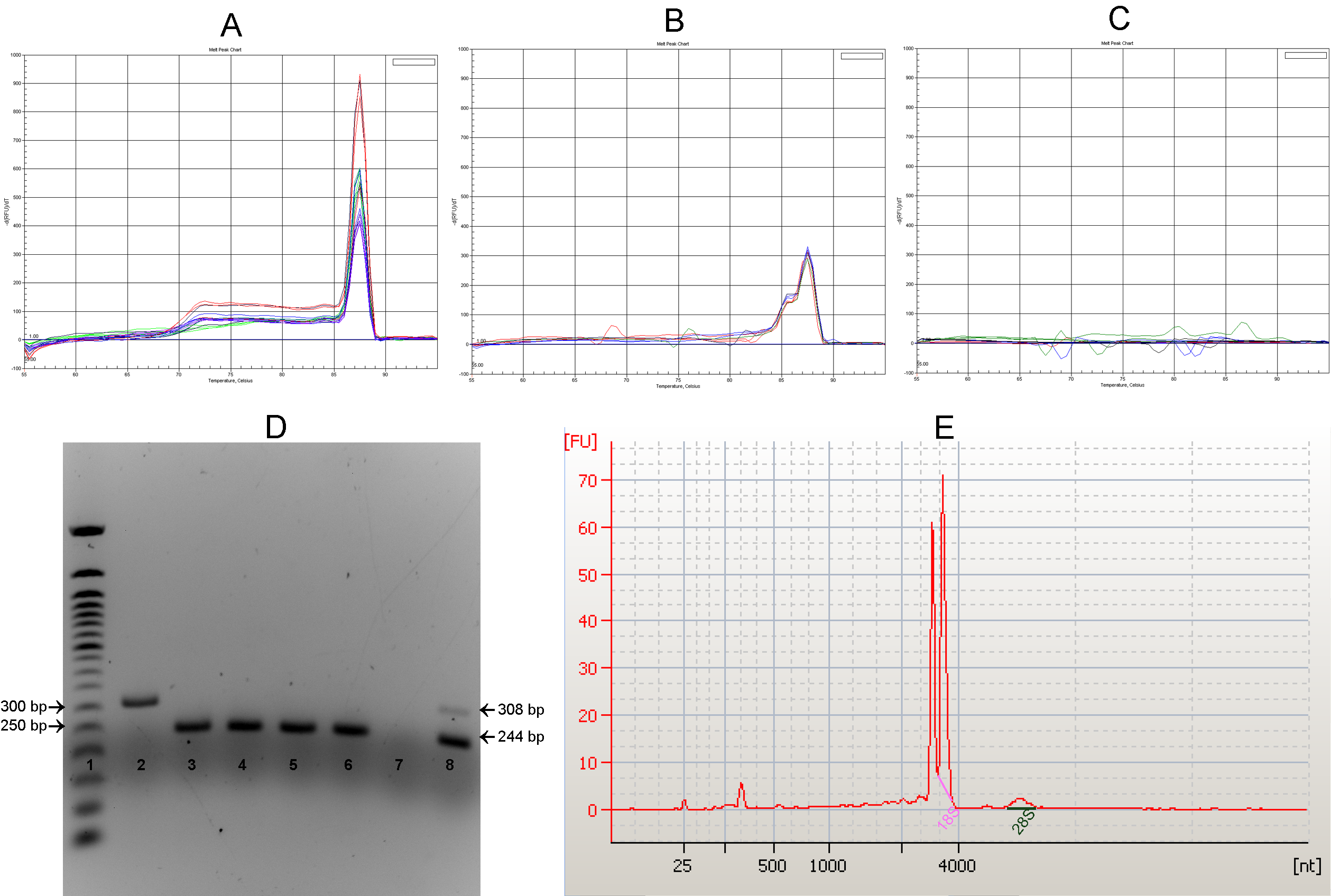

Supplement: Figure S1 — Quality control of RT and real-time PCR performance. (A) Representative melting curve for real-time PCR products exhibits only one symmetrical peak. (B) An asymmetric melting curve suggesting unspecific amplification. These types of primer sets were not used for analyses. (C) Absence of a melting peak in no-template-control (NTC) reactions suggesting an absence of primer-dimers during real-time PCR reaction. (D) Agarose gel electrophoresis to verify PCR specificity of the tested primer set and confirm the absence of potential genomic DNA contamination in cDNA samples. The representative data were from the RpII215-specific primer set predicted to generate a 308 bp fragment from genomic DNA and a 244 bp fragment from cDNA. Lanes from left to right are: 1, 50 bp DNA ladder (#N3236S, NEB); 2, a genomic DNA sample; 3–6, representative cDNA samples used in our experiments; 7, a negative control without adding M-MLV reverse transcriptase during RT; 8, a cDNA sample without DNase 1 pre-treatment. (E) The integrity of the representative RNA samples were assessed using Agilent 2100 Bioanalyzer. The electropherogram shows 2 sharp rRNA peaks that migrate close to each other, representing a typical Drosophila ribosomal RNA profile and suggesting excellent integrity of the RNA sample. (TIF) [file pone.0017762.s001.tif]
