# Supplementary material for: Robust RT-qPCR Data Normalization: Validation and Selection of Internal Reference Genes during Post-Experimental Data Analysis
Source: PLoS One. 2011 Mar 15;6(3):e17762. doi: 10.1371/journal.pone.0017762 (PMC3058000; doi:10.1371/journal.pone.0017762)
Supplement: Table S1 — Classic reference genes unsuitable for brain aging and neurodegeneration study in Drosophila *. (DOC) [file pone.0017762.s002.doc]

| **Table S1. Classic reference genes unsuitable for brain aging and neurodegeneration study in *Drosophila**** | | | | | |
| --- | --- | --- | --- | --- | --- |
| **Human genes** | |  | **Dr. homologues** | | **References** |
| **Symbol** | **Name** |  | **Symbol** | **Locus ID** |
| *ACTB* | β-actin |  | *Act88F* ^b^ | CG5178 | 1-5 |
| *B2M* | β-2-microglobulin |  | – ^a^ |  | 4-6 |
| *HGPRT* | Hypoxanthine-guanine phosphoribosyl transferase |  | – ^a^ |  | 3-5 |
| *Alb* | Albumin |  | – ^a^ |  | 4 |
| *TBP* | TATA binding protein |  | *Tbp* ^b^ | CG9874 | 2, 4-6 |
| *PPIA* | Peptidyl prolyl isomerase A |  | *CG7768* ^b^ | CG7768 | 4 |
| *PLA* | Phospholipase A2 |  | – ^a^ |  | 7 |
| *G6PDH* | Glucose-6-phosphate dehydrogenase |  | *Zw* ^b^ | CG12529 | 7 |
| *RPS18* | ribosomal protein S18 |  | *RpS18* ^b^ | CG8900 | 6 |
| *UBC* | Ubiquitin C |  | *Ubi-p63E* ^c^ | CG11624 | 5, 6, 8-10 |
| * The unsuitability is due to a) No *Drosophila* homologous genes, b) Low expression in *Drosophila* brain (FlyAtlas levels<100) or c) Highly repetitive mRNA sequence. In addition, the 18S or 28S rRNA transcripts are highly abundant and have been recommended to be excluded from reference candidates[5]. | | | | | |

# Supporting References

1. Van Hiel MB, Van Wielendaele P, Temmerman L, Van Soest S, Vuerinckx K, Huybrechts R, Broeck JV, Simonet G. Identification and validation of housekeeping genes in brains of the desert locust Schistocerca gregaria under different developmental conditions. *BMC Molecular Biology* 2009, 10:56.

2. Scharlaken B, de Graaf DC, Goossens K, Brunain M, Peelman LJ, Jacobs FJ. Reference gene selection for insect expression studies using quantitative real-time PCR: The head of the honeybee, Apis mellifera, after a bacterial challenge. *Journal of Insect Science* 2008, 8(33):1-10.

3. Huggett J, Dheda K, Bustin S, Zumla A. Real-time RT-PCR normalisation; strategies and considerations. *Genes and Immunity* 2005, 6(4):279-284.

4. Radonic A, Thulke S, Mackay IM, Landt O, Siegert W, Nitsche A. Guideline to reference gene selection for quantitative real-time PCR. *Biochemical and Biophysical Research Communications* 2004, 313(4):856-862.

5. Vandesompele J, De Preter K, Pattyn F, Poppe B, Van Roy N, De Paepe A, Speleman F. Accurate normalization of real-time quantitative RT-PCR data by geometric averaging of multiple internal control genes. *Genome Biology* 2002, 3(7):RESEARCH0034.

6. Coulson DT, Brockbank S, Quinn JG, Murphy S, Ravid R, Irvine GB, Johnston JA. Identification of valid reference genes for the normalization of RT qPCR gene expression data in human brain tissue. *BMC Molecular Biology* 2008, 9:46.

7. Dheda K, Huggett JF, Bustin SA, Johnson MA, Rook G, Zumla A. Validation of housekeeping genes for normalizing RNA expression in real-time PCR. *BioTechniques* 2004, 37(1):112-114, 116, 118-119.

8. Cikos S, Bukovska A, Koppel J. Relative quantification of mRNA: comparison of methods currently used for real-time PCR data analysis. *BMC Molecular Biology* 2007, 8:113.

9. Chintapalli VR, Wang J, Dow JA. Using FlyAtlas to identify better Drosophila melanogaster models of human disease. *Nature Genetics* 2007, 39(6):715-720.

10. Suzuki T, Higgins PJ, Crawford DR. Control selection for RNA quantitation. *BioTechniques* 2000, 29(2):332-337.
