# Supplementary material for: Robust RT-qPCR Data Normalization: Validation and Selection of Internal Reference Genes during Post-Experimental Data Analysis
Source: PLoS One. 2011 Mar 15;6(3):e17762. doi: 10.1371/journal.pone.0017762 (PMC3058000; doi:10.1371/journal.pone.0017762)
Supplement: Table S2 — Sequences and PCR efficiencies of primer sets. (DOC) [file pone.0017762.s003.doc]

| **Table S2. Sequences and PCR efficiencies of primer sets** | | | |
| --- | --- | --- | --- |
| Gene | Primer sequences (5'→3') | R^2^ | E * |
| **22 candidate reference genes** | | | |
| *Gapdh2* | CGTTCATGCCACCACCGCTA; CCACGTCCATCACGCCACAA | 0.9997 | 99.96% |
| *αTub84B* | TGGGCCCGTCTGGACCACAA; TCGCCGTCACCGGAGTCCAT | 0.9993 | 99.96% |
| *RpL32* | AAGCGGCGACGCACTCTGTT; GCCCAGCATACAGGCCCAAG | 0.9990 | 99.98% |
| *RpL13A* | AGCTGAACCTCTCGGGACAC; TGCCTCGGACTGCCTTGTAG | 0.9996 | 99.91% |
| *Ef1a48D* | TCCTCCGAGCCACCATACAG; GTCTTGCCGTCAGCGTTACC | 0.9987 | 100% |
| *eIF-1A* | ATCAGCTCCGAGGATGACGC; GCCGAGACAGACGTTCCAGA | 0.9997 | 99.21% |
| *Sdha* | CATGCTGCTGTGTTCCGCGA; ACCATCCAGGGGCTTGCTGA | 0.9966 | 99.36% |
| *GstD1* | GACTCCCTGTACCCTAAGTGC; TCGGCTACGGTAAGGGAGTCA | 0.9974 | 99.60% |
| *Cyp1* | TCGGCAGCGGCATTTCAGAT; TGCACGCTGACGAAGCTAGG | 0.9991 | 99.82% |
| *14-3-3ε* | CATGAACGATCTGCCACCAAC; CTCTTCGCTCAGTGTATCCAAC | 0.9990 | 99.83% |
| *Exba* | ACTTCTCGCAGCGCACCAAC; TCGAGGCGAAAGCCTGCAAC | 0.9990 | ***92.93%*** |
| *Act5C* | GGCGCAGAGCAAGCGTGGTA; GGGTGCCACACGCAGCTCAT | 0.9996 | 100% |
| *Su(Tpl)* | TCCCAGAGCCACCGTTACAC; CTGGTTGCAGGCGTTTAGCGT | 0.9990 | 99.98% |
| *Faf* | GGAACAAGCTCGACGCGATG; GGCGAATCGGGATGGCAGAA | 0.9972 | ***83.44%*** |
| *CG13220* | TGGTGAGCTACGGAGCCCTTG; GGGGCCTGCCGTAAATGTAGA | 0.9994 | 99.96% |
| *Robl* | AGCGGTAGTGTCTGCCGTGT; CCAGCGTGGATTTGACCGGA | 0.9995 | 99.96% |
| *Rap2l* | ACTTCCGTGCATTACGTGCG; CCGACCCGAGCACAACAACT | 0.9995 | 99.98% |
| *l(3)02640* | AGCGAGAAGATGCGCGGGAC; GGCTGTGCGACGAAGAGATGA | 0.9990 | 99.09% |
| *RpII215* | GACTGGTGGTTCGGCCAAGA; TGTCAGGCCGGCAAGATACG | 0.9992 | 99.98% |
| *nrv2* | TCGAATGACTTGCCCGCGAA; GCCCTCGCACGATACCCAAA | 0.9992 | 99.98% |
| *Elav* | CGCGACAAGTCGCAGGTCTAC; TCATCGTCTTTGGCAGCCCCG | 0.9981 | 97.99% |
| *Appl* | AGTTCCCGAGGGCTGTCTGTT; GCTTGGGACAGCAGACGAACT | 0.9989 | 99.96% |
| **7 target genes** | |  |  |
| *Atg1* | TCTGTGCGGCTCTCCCATGT; GAATGGCGCCTTGCCAGTCA | 0.9993 | 99.16% |
| *Rab5* | AGTCCGCTGTGGGCAAGTC; CTCCTGGTACTCGTGGAACTGTC | 0.9997 | 99.98% |
| *Lamp1* | GGAGCGACGGAGCGTTTAAG; CGCAATCGTGTCCAACTGAGA | 0.9993 | 98.49% |
| *CathD* | GGGAATGAGATCGCAAGAAAC; CCTACTGGCAAATCAAGATGG | 0.9995 | 96.17% |
| *InR* | GCGGGATACGGCGATTTCAC; AACGATCAGGAACGCTAGGC | 0.9987 | 99.98% |
| *Ire1* | CCACGGTCTCTGATGAGGCTTAC; TCTTGGTTGCTGTTCTGGTCTCC | 0.9996 | 97.29% |
| *Hsp70* | GGACAACCAGCCCGGAGTCT; GCAGGTGGAATGCCGGACAGA | 0.9991 | 99.90% |
| * PCR efficiency (E) was calculated as described [Pfaffl MW, Tichopad A, Prgomet C, Neuvians TP. Determination of stable housekeeping genes, differentially regulated target genes and sample integrity: BestKeeper--Excel-based tool using pair-wise correlations*. Biotechnology Letter*s 2004, 26(6):509-515]. | | | |
