# Supplementary material for: Robust RT-qPCR Data Normalization: Validation and Selection of Internal Reference Genes during Post-Experimental Data Analysis
Source: PLoS One. 2011 Mar 15;6(3):e17762. doi: 10.1371/journal.pone.0017762 (PMC3058000; doi:10.1371/journal.pone.0017762)
Supplement: Table S3 — Experimental details of fly head samples. (DOC) [file pone.0017762.s004.doc]

| **Table S3. Experimental details of fly head samples** | | | |
| --- | --- | --- | --- |
| **Sample** | **Genotype** | **T (ºC)** | **Age (days)** |
| **Standard samples** | | | |
| Standard | *w^1118^* | 25 | 10 |
|  |  |  |  |
| **Aging-related samples: Flies at different ages or treated with aging-related stresses** | | | |
| Control | *w^1118^* | 25 | 10 |
| Mid-age | *w^1118^* | 25 | 30 |
| Old-age | *w^1118^* | 25 | 50 |
| Low-T | *w^1118^* | 18 | 10 |
| High-T | *w^1118^* | 32 | 10 |
| Male * | *w^1118^* | 25 | 10 |
| Heat-shocked † | *w^1118^* | 25 | 10 |
| Instant starvation ‡ | *w^1118^* | 25 | 10 |
| Oxidative stress # | *w^1118^* | 25 | 10 |
|  |  |  |  |
| **Neurodegeneration-related samples: Flies have or have not expression of Aβ_1-42_/tau in neurons** | | | |
| Control, 3 days | *elav-Gal4/+;+;+* | 28 | 3 |
| Aβ_1-42_, 3 days | *elav-Gal4/+;UAS-Aβ_1-42_/+;+* | 28 | 3 |
| tau, 3 days | *elav-Gal4/+;+;UAS-tau^R406W^/+* | 28 | 3 |
| Control, 20 days | *elav-Gal4/+;+;+* | 28 | 20 |
| Aβ_1-42_, 20 days | *elav-Gal4/+;UAS-Aβ_1-42_/+;+* | 28 | 20 |
| tau, 20 days | *elav-Gal4/+;+;UAS-tau^R406W^/+* | 28 | 20 |
| * All samples are from females except the male sample;  † Flies incubated at 37ºC for 2 hours before RNA extraction;  ‡ Flies incubated in empty vials (with wet filter paper) for 12hrs before RNA extraction.  # Flies incubated in vials with 1% H_2_O_2_ and Schneider's medium for 48h. Fresh H_2_O_2_/Schneider's added every 8h. | | | |
