# Supplementary material for: Robust RT-qPCR Data Normalization: Validation and Selection of Internal Reference Genes during Post-Experimental Data Analysis
Source: PLoS One. 2011 Mar 15;6(3):e17762. doi: 10.1371/journal.pone.0017762 (PMC3058000; doi:10.1371/journal.pone.0017762)
Supplement: Table S4 — Relative expression of target genes in aging-related samples normalized by different subsets of reference genes. (DOC) [file pone.0017762.s005.doc]

| **Table S4. Relative expression of target genes in aging-related samples normalized by different subsets of reference genes** | | | | | | | | | | | |
| --- | --- | --- | --- | --- | --- | --- | --- | --- | --- | --- | --- |
| **Sample Name *** | **Expression Ratios Normalized by** | | | | | | | | | | |
|  | **1 Ref. Gene** | | |  | **3 Ref. Genes** | | |  | **13 Ref. Genes** | | |
|  | **Mean** | **SEM** | **P **** |  | **Mean** | **SEM** | **P** |  | **Mean** | **SEM** | **P** |
| ***Atg1*** | | | | | | | | | | | |
| CTL | 1.000 | 0.120 | Ref. |  | 1.000 | 0.090 | Ref. |  | 1.000 | 0.081 | Ref. |
| A30 | 1.040 | 0.064 | 0.670 |  | 0.926 | 0.053 | 0.442 |  | 0.873 | 0.046 | 0.196 |
| A50 | 1.220 | 0.091 | 0.055 |  | 0.985 | 0.067 | 0.887 |  | 0.884 | 0.057 | 0.274 |
| L18 | 0.960 | 0.094 | 0.694 |  | 0.828 | 0.073 | 0.109 |  | 0.787 | 0.059 | 0.054 |
| H32 | 0.982 | 0.091 | 0.862 |  | 0.906 | 0.073 | 0.374 |  | 0.808 | 0.056 | 0.087 |
| HSK | 0.718 | 0.090 | 0.017 |  | 0.659 | 0.072 | 0.006 |  | 0.661 | 0.068 | 0.006 |
| OXI | 1.397 | 0.163 | 0.042 |  | 1.179 | 0.134 | 0.318 |  | 1.193 | 0.130 | 0.284 |
| STA | 0.971 | 0.104 | 0.741 |  | 0.877 | 0.053 | 0.179 |  | 0.883 | 0.037 | 0.199 |
| ***CathD*** | | | | | | | | | | | |
| CTL | 1.000 | 0.112 | Ref. |  | 1.000 | 0.079 | Ref. |  | 1.000 | 0.069 | Ref. |
| A30 | 1.136 | 0.069 | 0.136 |  | 1.011 | 0.056 | 0.894 |  | 0.953 | 0.049 | 0.584 |
| A50 | 1.133 | 0.077 | 0.133 |  | 0.915 | 0.057 | 0.320 |  | 0.820 | 0.048 | 0.052 |
| L18 | 0.818 | 0.083 | 0.057 |  | 0.705 | 0.064 | 0.006 |  | 0.670 | 0.053 | 0.003 |
| H32 | 0.827 | 0.105 | 0.151 |  | 0.762 | 0.091 | 0.059 |  | 0.680 | 0.076 | 0.017 |
| HSK | 0.945 | 0.220 | 0.772 |  | 0.868 | 0.194 | 0.492 |  | 0.870 | 0.192 | 0.499 |
| OXI | 0.905 | 0.128 | 0.509 |  | 0.764 | 0.106 | 0.120 |  | 0.773 | 0.104 | 0.133 |
| STA | 1.043 | 0.118 | 0.588 |  | 0.942 | 0.054 | 0.466 |  | 0.948 | 0.030 | 0.515 |
| ***Hsp70*** | | | | | | | | | | | |
| CTL | 1.000 | 0.119 | Ref. |  | 1.000 | 0.082 | Ref. |  | 1.000 | 0.070 | Ref. |
| A30 | 1.759 | 0.330 | 0.047 |  | 1.566 | 0.292 | 0.121 |  | 1.475 | 0.273 | 0.184 |
| A50 | 2.023 | 0.169 | 0.000 |  | 1.633 | 0.129 | 0.001 |  | 1.465 | 0.111 | 0.007 |
| L18 | 1.662 | 0.253 | 0.016 |  | 1.432 | 0.209 | 0.084 |  | 1.361 | 0.189 | 0.139 |
| H32 | 4.057 | 0.515 | 0.000 |  | 3.741 | 0.445 | 0.000 |  | 3.336 | 0.373 | 0.001 |
| HSK | 223.851 | 44.035 | 0.000 |  | 205.639 | 38.293 | 0.000 |  | 206.136 | 37.586 | 0.000 |
| OXI | 1.235 | 0.130 | 0.136 |  | 1.043 | 0.106 | 0.773 |  | 1.055 | 0.102 | 0.711 |
| STA | 0.759 | 0.098 | 0.019 |  | 0.685 | 0.047 | 0.005 |  | 0.690 | 0.030 | 0.006 |
| ***InR*** | | | | | | | | | | | |
| CTL | 1.000 | 0.154 | Ref. |  | 1.000 | 0.133 | Ref. |  | 1.000 | 0.127 | Ref. |
| A30 | 1.695 | 0.071 | 0.000 |  | 1.510 | 0.051 | 0.003 |  | 1.422 | 0.038 | 0.009 |
| A50 | 2.126 | 0.196 | 0.000 |  | 1.717 | 0.151 | 0.003 |  | 1.540 | 0.132 | 0.013 |
| L18 | 0.804 | 0.070 | 0.165 |  | 0.693 | 0.052 | 0.041 |  | 0.658 | 0.040 | 0.026 |
| H32 | 1.173 | 0.097 | 0.243 |  | 1.082 | 0.075 | 0.571 |  | 0.965 | 0.055 | 0.804 |
| HSK | 1.067 | 0.150 | 0.690 |  | 0.980 | 0.123 | 0.905 |  | 0.982 | 0.117 | 0.916 |
| OXI | 2.506 | 0.315 | 0.001 |  | 2.115 | 0.258 | 0.004 |  | 2.140 | 0.250 | 0.004 |
| STA | 1.099 | 0.144 | 0.534 |  | 0.992 | 0.087 | 0.960 |  | 0.999 | 0.074 | 0.994 |
| ***Ire1*** | | | | | | | | | | | |
| CTL | 1.000 | 0.175 | Ref. |  | 1.000 | 0.156 | Ref. |  | 1.000 | 0.151 | Ref. |
| A30 | 1.664 | 0.094 | 0.002 |  | 1.482 | 0.076 | 0.015 |  | 1.396 | 0.065 | 0.037 |
| A50 | 1.862 | 0.211 | 0.002 |  | 1.503 | 0.165 | 0.036 |  | 1.348 | 0.145 | 0.124 |
| L18 | 0.840 | 0.085 | 0.338 |  | 0.724 | 0.066 | 0.114 |  | 0.688 | 0.055 | 0.079 |
| H32 | 0.846 | 0.060 | 0.378 |  | 0.780 | 0.040 | 0.218 |  | 0.696 | 0.018 | 0.100 |
| HSK | 0.720 | 0.112 | 0.132 |  | 0.662 | 0.094 | 0.076 |  | 0.663 | 0.091 | 0.077 |
| OXI | 1.601 | 0.295 | 0.082 |  | 1.351 | 0.245 | 0.281 |  | 1.367 | 0.243 | 0.262 |
| STA | 1.113 | 0.200 | 0.606 |  | 1.005 | 0.152 | 0.981 |  | 1.012 | 0.145 | 0.956 |
| ***Lamp1*** | | | | | | | | | | | |
| CTL | 1.000 | 0.123 | Ref. |  | 1.000 | 0.094 | Ref. |  | 1.000 | 0.086 | Ref. |
| A30 | 1.218 | 0.062 | 0.041 |  | 1.085 | 0.048 | 0.383 |  | 1.022 | 0.040 | 0.819 |
| A50 | 1.571 | 0.104 | 0.000 |  | 1.268 | 0.076 | 0.027 |  | 1.137 | 0.064 | 0.216 |
| L18 | 1.001 | 0.094 | 0.996 |  | 0.862 | 0.072 | 0.201 |  | 0.820 | 0.057 | 0.103 |
| H32 | 1.163 | 0.122 | 0.228 |  | 1.073 | 0.102 | 0.580 |  | 0.957 | 0.082 | 0.739 |
| HSK | 0.773 | 0.140 | 0.124 |  | 0.710 | 0.121 | 0.057 |  | 0.712 | 0.118 | 0.059 |
| OXI | 1.056 | 0.100 | 0.653 |  | 0.892 | 0.080 | 0.393 |  | 0.902 | 0.076 | 0.438 |
| STA | 1.000 | 0.136 | 0.998 |  | 0.903 | 0.092 | 0.432 |  | 0.909 | 0.084 | 0.460 |
| ***Rab5*** | | | | | | | | | | | |
| CTL | 1.000 | 0.108 | Ref. |  | 1.000 | 0.073 | Ref. |  | 1.000 | 0.062 | Ref. |
| A30 | 1.161 | 0.067 | 0.061 |  | 1.034 | 0.054 | 0.669 |  | 0.973 | 0.046 | 0.734 |
| A50 | 1.292 | 0.113 | 0.011 |  | 1.044 | 0.087 | 0.654 |  | 0.936 | 0.075 | 0.512 |
| L18 | 1.008 | 0.091 | 0.919 |  | 0.869 | 0.069 | 0.125 |  | 0.826 | 0.054 | 0.050 |
| H32 | 0.973 | 0.082 | 0.738 |  | 0.897 | 0.064 | 0.224 |  | 0.800 | 0.047 | 0.030 |
| HSK | 0.960 | 0.107 | 0.661 |  | 0.882 | 0.081 | 0.209 |  | 0.884 | 0.074 | 0.217 |
| OXI | 1.346 | 0.141 | 0.033 |  | 1.136 | 0.114 | 0.355 |  | 1.149 | 0.110 | 0.312 |
| STA | 1.117 | 0.134 | 0.170 |  | 1.008 | 0.070 | 0.918 |  | 1.015 | 0.052 | 0.851 |
| The different subsets of reference genes are described in the legend to Fig.4.  * CTL = Control; A30 = Mid-age; A50 = Old-age; L18 = Low-T; H32 = High-T; HSK = Heat-shocked; OXI = Oxidative stress; STA = Instant starvation. See Table S3 for additional details.  ** P values are two-tailed and obtained by Student's t-test. | | | | | | | | | | | |
