# Supplementary material for: Robust RT-qPCR Data Normalization: Validation and Selection of Internal Reference Genes during Post-Experimental Data Analysis
Source: PLoS One. 2011 Mar 15;6(3):e17762. doi: 10.1371/journal.pone.0017762 (PMC3058000; doi:10.1371/journal.pone.0017762)
Supplement: Table S5 — Relative expression of target genes in neurodegeneration-related samples normalized by different subsets of reference genes. (DOC) [file pone.0017762.s006.doc]

| **Table S5. Relative expression of target genes in neurodegeneration-related samples normalized by different subsets of reference genes** | | | | | | | |
| --- | --- | --- | --- | --- | --- | --- | --- |
| Sample Name * | Expression Ratios Normalized by | | | | | | |
|  | 1 Ref. Gene | | |  | 3 Ref. Genes | | |
|  | Mean | SEM | P ** |  | Mean | SEM | P |
| ***Atg1*** | | | | | | | |
| C20 | 1.000 | 0.054 | Ref. |  | 1.000 | 0.059 | Ref. |
| A20 | 1.149 | 0.097 | 0.157 |  | 1.150 | 0.078 | 0.156 |
| T20 | 1.069 | 0.114 | 0.518 |  | 1.076 | 0.118 | 0.481 |
| ***CathD*** | | | | | | | |
| C20 | 1.000 | 0.206 | Ref. |  | 1.000 | 0.208 | Ref. |
| A20 | 1.014 | 0.079 | 0.954 |  | 1.014 | 0.057 | 0.953 |
| T20 | 0.985 | 0.071 | 0.945 |  | 0.991 | 0.076 | 0.967 |
| ***Hsp70*** | | | | | | | |
| C20 | 1.000 | 0.124 | Ref. |  | 1.000 | 0.127 | Ref. |
| A20 | 1.189 | 0.099 | 0.233 |  | 1.189 | 0.080 | 0.232 |
| T20 | 1.558 | 0.167 | 0.012 |  | 1.567 | 0.173 | 0.011 |
| ***InR*** | | | | | | | |
| C20 | 1.000 | 0.117 | Ref. |  | 1.000 | 0.119 | Ref. |
| A20 | 1.444 | 0.217 | 0.123 |  | 1.444 | 0.205 | 0.123 |
| T20 | 1.446 | 0.141 | 0.018 |  | 1.454 | 0.147 | 0.017 |
| ***Ire1*** | | | | | | | |
| C20 | 1.000 | 0.037 | Ref. |  | 1.000 | 0.044 | Ref. |
| A20 | 1.485 | 0.092 | 0.000 |  | 1.485 | 0.055 | 0.000 |
| T20 | 1.200 | 0.094 | 0.015 |  | 1.207 | 0.099 | 0.013 |
| ***Lamp1*** | | | | | | | |
| C20 | 1.000 | 0.107 | Ref. |  | 1.000 | 0.110 | Ref. |
| A20 | 0.898 | 0.107 | 0.517 |  | 0.898 | 0.097 | 0.518 |
| T20 | 0.714 | 0.101 | 0.062 |  | 0.719 | 0.103 | 0.066 |
| ***Rab5*** | | | | | | | |
| C20 | 1.000 | 0.120 | Ref. |  | 1.000 | 0.122 | Ref. |
| A20 | 0.894 | 0.107 | 0.526 |  | 0.894 | 0.097 | 0.527 |
| T20 | 0.952 | 0.086 | 0.725 |  | 0.957 | 0.090 | 0.756 |
| The 1 reference gene is *Gapdh2*; the 3 reference genes are *Gapdh2*, *RpL13A* and *l(3)02640*. The reference genes are selected based on their rank order of expression stability among the 15 candidate reference genes in the 3 aged neurodegeneration-related samples (Fig.2B, red line).  * C20 = Control, 20 days; A20 = Aβ_1-42_, 20 days; T20 = tau, 20 days. See Table S3 for details.  ** P values are two-tailed and obtained by Student's t-test. | | | | | | | |
